# Conditional Survival of female patients with operable invasive Breast Cancer in US: A population-based study

**DOI:** 10.7150/jca.46183

**Published:** 2020-07-31

**Authors:** Bolun Ai, Xiangyu Wang, Xiangyi Kong, Zhongzhao Wang, Yi Fang, Jing Wang

**Affiliations:** Department of Breast Surgical Oncology, China National Cancer Center/Cancer Hospital, Chinese Academy of Medical and Peking Union Medical College, No. 17 Panjiayuan-Nanli, Chaoyang District, Beijing 100021, P.R. China.

**Keywords:** Invasive breast cancer, Disease-specific survival, Conditional survival, SEER database

## Abstract

**Background:** Conditional survival (CS) is used to describe the dynamic possibility of survival, considering the changes of death risk with time lapsing. This study aimed to estimate the conditional disease-specific survival (CDS) for the female with operable invasive breast cancer.

**Methods:** The data was obtained from Surveillance, Epidemiology, and End Result Program of the National Cancer Institute. The hazard rate was calculated using kernel density smoothing method. The disease-specific survival (DSS) rates were estimated and compared using Kaplan-Meier method and log-rank test. The Cox regression model was used to adjust confounding factors. The CDS was calculated by CDS(*y|x*)=DSS(*x + y*)/DSS(*x*), where DSS(*x*) representatives the DSS at *x* year.

**Results:** The 5-year, 10-year, and 15-year DSS was 88.7%, 82.0%, and 78.3%, respectively. The hazard rate after surgery increased initially and peaked at about 1.5 years, then decreased gradually. Meanwhile, the CDS decreased just after surgery then increased continuously, which showed a contrary trend with hazard rate. Patients with high risk factors had greater survival gap between cumulative DSS and CDS. The changing trend of CDS in patients with high risk factors was more significant, and the CDS gap between low-risk patients and high-risk patients gradually decreased over time.

**Conclusion:** CS could provide a more precise long-term prognostic evaluation compared to traditional cumulative survival, especially for long-time survivors with high risk.

## Introduction

Breast cancer is the most common malignant tumor among women and the leading cause of cancer-related deaths among women in the world. Recent results showed that approximately 270,000 newly diagnosed cases and more than 40,000 deaths in the United States 1, 2. Patients with invasive breast carcinoma achieve better prognosis than those diagnosed a few decades ago, mainly because the multidisciplinary treatment performed 2-4.

When estimating the long-term prognosis, the traditional cumulative survival rates after surgery are usually reported. However, this method has its limitations, especially for long-term survivors. For example, it could only reflect a constant hazard rate and calculated survival rate from the initial follow-up. However, the survival rate of patients may change with survival time prolonged, since the death risk or hazard ratio changed.

Conditional survival (CS), which considers the changing death risk with prolonged survival time, is a method to estimate the dynamic possibility of long-term survival 5. The CS had been reported in many types of cancers, and has provided accurate and valuable prognostic information for cancer survivors and oncologists 6-10. However, to our knowledge, only few studies have assessed CS of breast cancer, and no studies have reported the CS of patients with operable invasive breast carcinoma 6, 7, 11. Additionally, no studies have evaluated the CS of patients based on the hormone receptor and human epidermal growth factor receptor 2 (Her2) expression status, which could provide more precise information for clinical decisions.

Thus, in the current study, we provide a descriptive analysis of the CS for female with operable invasive breast carcinoma from 1998 to 2015 in the United States.

## Materials and Methods

**Figure 1:**
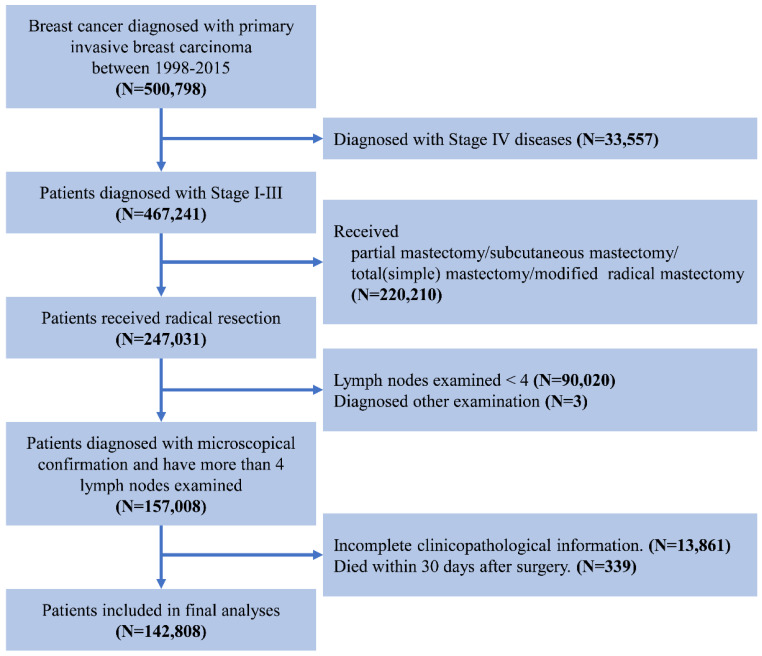
The flowchart of data selection for Surveillance, Epidemiology, and End Results patient data set.

We used data from Surveillance, Epidemiology, and End Result (SEER) database of the National Cancer Institute, which covers about 26% of the United States 12. The study population included female patients with primary operable invasive breast carcinoma between January 1998 and December 2015. The exclusion criterion was as follows: 1) with other malignancy histories; 2) no surgical treatment; 3) without microscopical diagnostic confirmation; 4) the number of examined lymph node < 4; 5) incomplete follow-up information or pathological data; 6) died within one month after surgery. The screening process was showed in **Figure 1.** The characteristics of the patients were analyzed in the current study including age at diagnosis, race, tumor grade, the estrogen receptor (ER) status, progesterone receptor (PR) status, Her2 status, AJCC TNM Staging System, survival months and survival status 13. Age was divided into four groups: ≤50, 51-60, 61-70, and ≥70 years. The breast subtype was also divided into 4 subtypes according to the hormone receptor and Her2 Status. Since SEER is a public database, no need for informed consent and ethical consent.

### Statistical Analysis

Continuous variables are presented as mean ± standard deviation (SD) values. Categorical variables are expressed as counts and proportions. The hazard rate function was calculated using kernel density smoothing method. The Disease-specific survival (DSS) rate was calculated based on the variables “survival months” and “SEER cause-specific death classification” and using the Kaplan-Meier method. The differences of DSS were compared with the log-rank test. Univariate and multivariate Cox proportional hazards regression model was applied to identify the independent factors associated with prognosis among clinicopathologic characteristics. Those variables with statistical significance in the univariate model were included in the multivariate analysis. Since the SEER database provided the Her2 expression status only with patients diagnosed after 2010, we also performed univariate and multivariate analyses for this group of patients separately.

CS, the probability that a patient who has survived *x* years will survive for another *y* years, was calculated by CS(*y|x*)=DSS(*x + y*)/DSS(*x*), where DSS(*x*) representatives the DSS at *x* year. In our current study, we estimated the 3-year conditional DSS (CDS3) of patients. For example, the possibility of the patients who have survived 1 year after operation remaining alive for an additional 3 years was expressed as CDS3(1), which equals to the DSS(4)/DSS(1). We also evaluated the CDS3 stratified by independent prognostic factors.

For all analyses, the significance test was based on a two-tailed *P* value < 0.05. All analyses were performed using IBM SPSS 22.0 (SPSS, Inc, Chicago, IL) and R software (version 3.2.1, R Foundation for Statistical Computing, Vienna, Austria).

## Results

### Clinicopathologic Characteristics

A total of 142,808 patients who met the criteria were included in our study. The clinicopathological characteristics of these patients were presented in **Table 1.** The median age was 59.05 ± 13.80 years. Grade II- IV patients accounted for more than 80% of total cohort. The number of patients with stage II was the most, followed by stage I and stage III. For hormone receptors (HR) status, more than 70% of patients were ER positive almost 60% patients were PR positive. Only 17.7% patients were Her2 positive. More than half of patients were classified as Her2-/HR+, and 13.6% patients were classified as triple-negative breast cancer.

### Cumulative DSS

**Figure 2:**
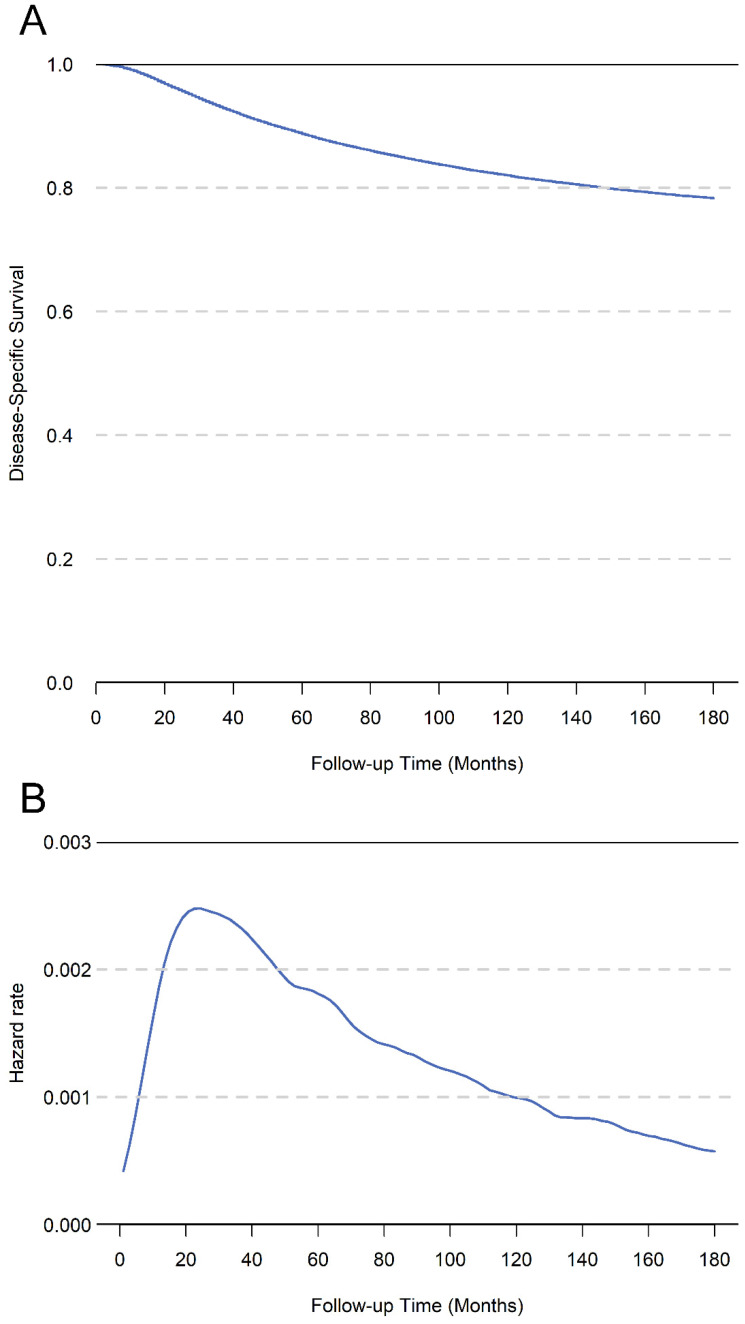
** A.** The cumulative disease-specific survival for total cohort patients.** B.** The hazard rate for total cohort patients.

The median survival time of the whole cohort was 89 months, and 21,787 patients (15.3%) had died at the last follow-up. The 5-year, 10-year, and 15-year DSS of total cohort was 88.7%, 82.0%, and 78.3%, respectively (**Figure 2A**). The hazard rate for patients increased just after surgery, peaked at about 1.5 years, and decreased thereafter (**Figure 2B**). In univariate analysis, the age, race, tumor grade, ER status, PR status, Her2 status and TNM stage were significantly associated with DSS (**Table 1**). In the multivariate analysis of total cohort, the results showed that age, race, tumor grade, ER status, PR status, and TNM stage were all independent prognostic factors. Additionally, we performed multivariate analysis only for patients diagnosed after 2010, the results showed age, tumor grade, ER status, PR status, Her2 status and TNM stage were independent prognostic factors (**Table 2**).

### CDS3 and Comparison with Cumulative DSS

**Figure 3:**
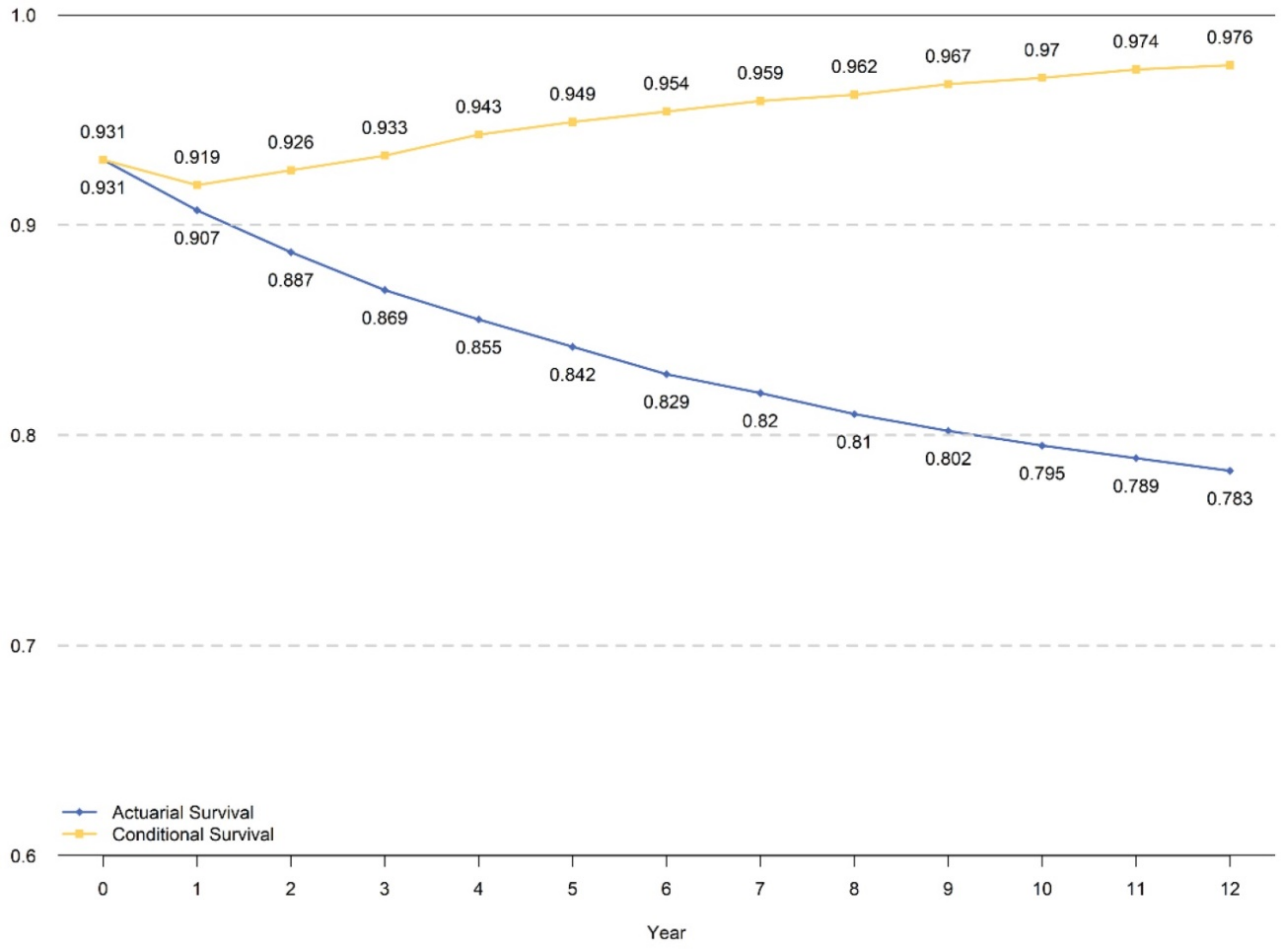
The cumulative DSS and CDS3 for total cohort patients.

The traditional cumulative DSS within 8 years and CDS3 for those who had already survived for 5 years are presented in **Figure 3.** The CDS3 decreased just after surgery in the first year then increased continuously, which has an inverse trend with hazard rate. The CDS3(1) (the probability of surviving to fourth years for patients who have already survived for 1 year was 91.9%, whereas the cumulative DSS at 4 years was 90.7%. Similarly, the CDS3 at 12 years (the probability of surviving to 15 years for patients who have already survived for 12 years) was 97.6% compared with a cumulative DSS at 12 years of 78.3%. From the 2^nd^ year, the CDS3 increased over time from 92.6% to 97.6%, whereas the cumulative DSS decreased from 88.7% to 78.3% at 15th years with time prolonging.

**Figure 4:**
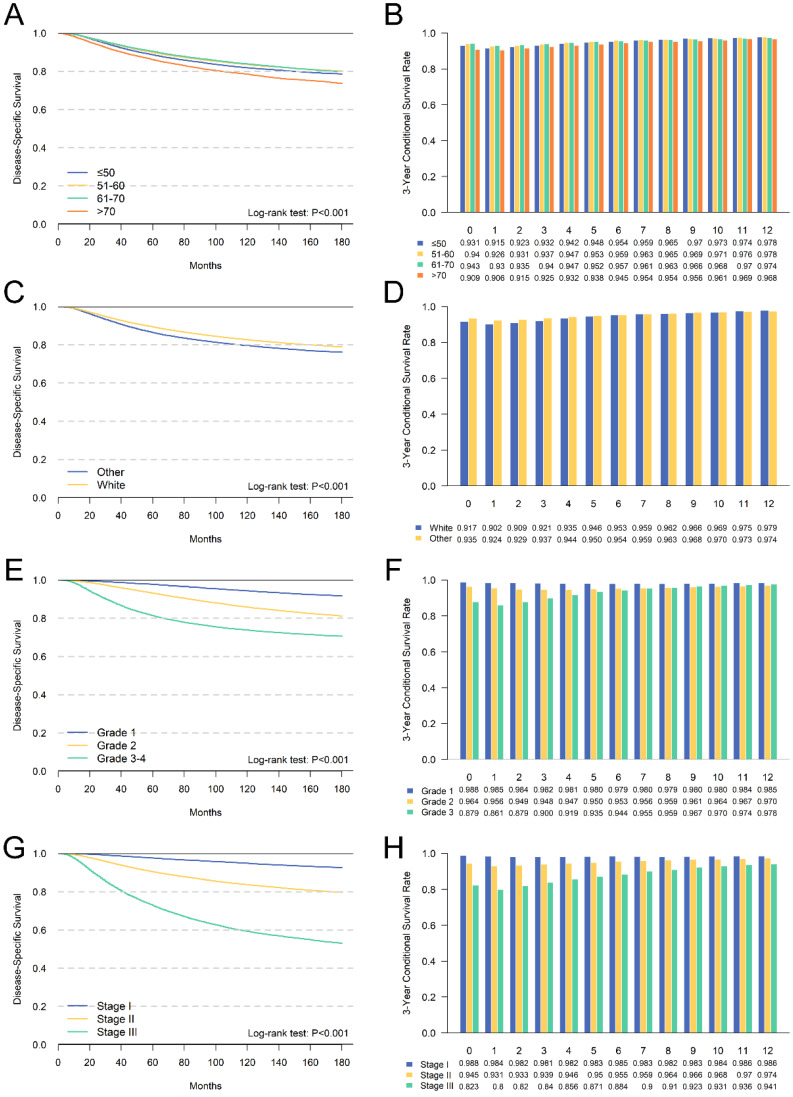
Comparison between cumulative DSS (A,C,E,G) and CDS3 (B,D,F,H) according to age (A,B), race (C,D), grade (E,F) and AJCC TNM stage (G,H).

**Figure 5:**
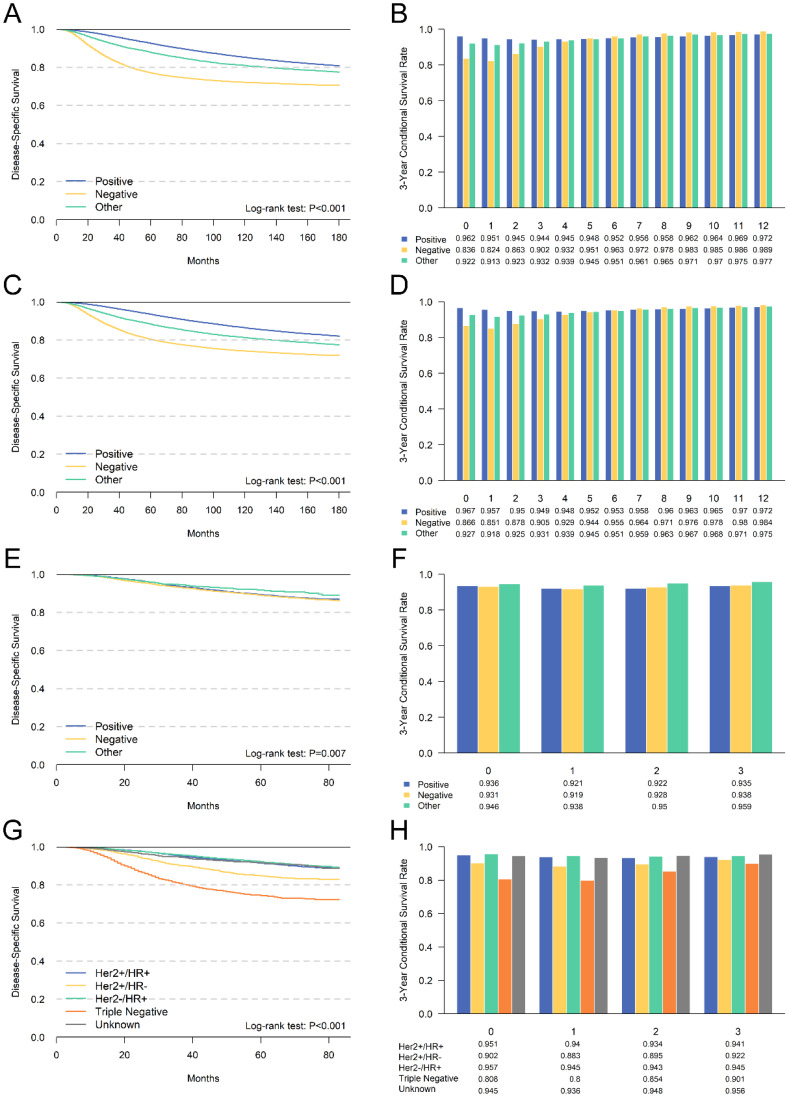
Comparison between cumulative DSS (A,C,E,G) and CDS3 (B,D,F,H) according to ER status (A,B), PR status (C,D), Her2 status(E,F) and breast cancer subtype (G,H).

We further performed a subgroup analysis of independent prognostic factors identified by multivariate analyses to assess its impact on cumulative DSS and CDS3. The cumulative DSS and CDS3 of different clinicopathological characteristics at different time points were presented in **Figures 4 and 5.** The cumulative DSS in each subgroup decreased with time elapsing, whereas the CDS3 of high-risk factors showing a trend of decreasing first and then increased. The CDS3 of low-risk factors changing trend is more gradual than that of high-risk. Furthermore, the survival rate gap between cumulative and conditional DSS was more significant among those patients with unfavorable tumor features. In contrast, patients with low-risk factors had a smaller survival gap. For example, patients with stage III had an 8-year DSS of 63.4% compared with a CDS3(5) of 87.1% (Δ = 23.7%), whereas patients with stage I had an 8-year DSS of 95.5% compared with a CDS3 at 5 years of 98.3% (Δ = 2.4%) (**Figure 4**). Besides, the changes in CS over time were more significant in patients who have high-risk features compared with those who have low-risk features. For example, patients with III-IV grade had larger changes (93.5%-86.1%; Δ = 7.4%) versus patients with grade I (98.8%-98.0%; Δ = 0.8%) (**Figure 4**). These patterns were also similar in other subgroups.

## Discussion

Patients with breast cancer were expected to have a better prognosis mainly because of the improved treatment and diagnostic procedures in recent years 14, 15. Invasive breast cancer was the most significant proportion of pathological types and standard surgical and multidisciplinary treatments have significantly improved the prognosis of these patients, however, the 5-year survival rate of patients with different pathological features still ranges from 54% to 98% 16, 17. When evaluating the prognosis, the traditional cumulative survival rate calculated from the surgery as the starting point for follow-up. However, cumulative survival rates only provide a constant hazard rate to assess the patients prognosis Actually, the survival is not only related to tumor characteristics, but also to survival time 18. The hazard rate of survival is not constant after surgery, and the survival probability at each time point is dynamic. Our results showed that the risk of postoperative mortality changes with the prolongation of survival time and the risk of death raised after surgery, peaked around 1.5 years, and then gradually decreased (**Figure 2B**). Therefore, cumulative survival may be too simple for accurately evaluating long-term outcomes, especially for patients have survived for a period.

Compared with cumulative survival, CS could reflect changes in survival probability due to prolonged survival, and is more useful for predicting prognosis 19-21. Donald et al. have reported the conditional survival of patients with different stages of breast cancer between 1983 and 1987 11. However, the staging and treatment plan was quite different and the prognosis was different from recent years. So far, there have been no reports of CS for invasive breast cancer based on clinicopathological characteristics. Therefore, we estimated the CS of invasive breast cancer who underwent radical surgery between 1998 and 2015. In order to ensure the accuracy of the staging, we only included patients with more than 4 lymph nodes retrieved.

The CS curve of the total cohort indicated that the prognosis was poor in the first two years after surgery, and the conditional survival rate also decreased. With the survival time prolonged, the CS increased gradually. These results suggested that the prognosis may change better. These results were consistent with the hazard rate curve. Additionally, it also showed that the CS may be more accurate in the evaluation of long-term survival than traditional cumulative survival.

We further identified independent risk factors for prognosis, including age, tumor grade, ER status, PR status, Her2 status and TNM stage. Meanwhile, we performed stratified analysis of CS for each factor. The results showed that CS was higher than DSS at all time points, and patients with high-risk factors had greater survival gap between cumulative DSS and CS. This finding was consistent with other malignancies 22-24. For example, patients with stage III disease had a survival gap of 23.7% between CDS3(5) and 8-year DSS (87.1 % - 63.4 %) compared with a 2.4% (98.3 % - 95.9 %) among those with stage I (**Figure 4**). Additionally, we found that CS in low-risk patients had a more stable trend, and the risk of death was almost unchanged. The changing trend of CS in high-risk patients was more significant, and the CS gap between low-risk patients and high-risk patients gradually decreased over time. For example, the variability of stage III (CDS3max-CDS3min = 87.1%-80.0% = 7.1%) was much larger than stage I (CDS3max-CDS3min = 0.7%). This may because the low-risk patients have better prognosis.

We evaluated the CS of hormone receptor status for the first time. We found that patients with ER negative or PR negative had the poorest prognosis. Meanwhile, CDS3 and DSS had the most substantial survival gap among patients with ER negative or PR negative. Additionally, we compared the survival difference between Her2 positive and negative patients using patients diagnosed after 2010. However, the survival difference was not significant, mainly due to the insufficient follow-up time. Additionally, the subtype of breast cancer according to the hormone receptor and Her2 receptor were also compared. The triple-negative breast cancer had the worst survival, and the CDS3 of these patients also changed most with the survival time increased. These results were consistent with other unfavorable clinicopathological features.

CS could provide more valuable information for making decision on individualized surveillance strategy. Since with the survival time prolongation, the risk of death decreased and the possibility of survival for additional time increased. Thus, a plateau or threshold value could be reached earlier for patients with low-risk, while for patients with high-risk may not reach this threshold in early postoperative period. Thus, dynamic CS estimates may play an essential role in designing an individualized surveillance strategy.

The conditional survival rate of tumors has been extensively studied in multiple cancers 25-29. But few studies have been conducted in breast cancer 30. Shui et al reported that the conditional survival rate of breast cancer based on the 3^th^ edition of the staging system, and found that patients with Stage IV had more significant changes in CS and larger survival rate gap between cumulative and conditional DSS over time 30. However, this research only focused on the 3th edition TNM staging system of 56,268 women who were diagnosed as having invasive breast cancer from 1983 to 1987. After decades, the treatment strategies and staging system have changed significantly, which have improved the prognosis of patients. Hence, in order to estimate the CS of breast cancer more accurately, we studied the impact of age, tumor grade, ER status, PR status, Her2 status and TNM stage on the conditional survival rate based on a larger sample size and more variables with comprehensive analysis. This study could provide a more accurate CS estimates for the patients with breast cancer from different point of view.

Our current study still had several limitations. First, our analysis was a retrospective study and the selection bias may be inherent. Second, we conducted stratified analyses based on the independent factors, however, other potential prognostic factors such as the economic status of patients and the treatment protocols may lead to some bias. Third, follow-up information regarding Her2 status is less than eight years, so the results of Her2 may not evaluate precisely. Future study with long follow-up time should be performed to elucidate this effect accurately. Fourth, our study did not include postoperative treatment in the analyses. Due to lack of detailed treatment information of chemotherapy, radiotherapy, and endocrine therapy in the SEER database, we could not evaluate the effect of the treatment on survival accurately, thus, future study with complete adjuvant treatment information could evaluate the CS of these treatment precisely. Fifth, all the experimental data presented and analyzed in the current study were downloaded from a publicly available database. We did not include our own clinical data in this present study, partly because the data size is not yet sufficient enough and the follow-up data is not yet long enough. However, as our data accumulated more and more, we will conduct a further analysis totally using the data from our hospital in the near future.

## Conclusion and Perspectives

In short, our study indicated that CS estimates could more accurately reflect the long-term survival probability of patients with operable invasive breast carcinoma. Especially for patients with high risk factors, as the survival time increases, the risk of death becomes lower. And this dynamic prognosis index may serve as a critical reference for follow-up strategy. In the future study, we would use the database from China to validate our results.

## Figures and Tables

**Table 1:** The Clinicopathologic characteristics of patients and the associations with DSS

Variables	No. (%)	5Y-DSS	*P* value
**Age**			<0.001
Mean ± SD	59.05 ± 13.80		
≤ 50	42,825 (30.0%)	88.6%	
51 - 60	36,065 (25.3%)	89.8%	
61 -70	31172 (21.8%)	90.4%	
> 70	32746 (22.9%)	86.1%	
**Race**			<0.001
Other	30426 (21.3%)	86.4%	
White	112382 (78.7%)	89.3%	
**Grade**			<0.001
I	22908 (16.0%)	97.7%	
II	57509 (40.3%)	93.1%	
III-IV	62391 (43.7%)	81.3%	
**Stage**			<0.001
I	45567 (31.9%)	97.6%	
II	63831 (44.7%)	90.4%	
III	33410 (23.4%)	72.8%	
**ER Status**			<0.001
Negative	32382 (22.7%)	77.1%	
Positive	100894 (70.7%)	92.6%	
Borderline/Unknown	9532 (6.7%)	87.8%	
**PR Status**			<0.001
Negative	46701 (32.7%)	93.4%	
Positive	84904 (59.5%)	80.3%	
Borderline/Unknown	11203 (7.9%)	88.3%	
Her2 Status*			0.007
Negative	32395 (77.6%)	88.9%	
Positive	7390 (17.7%)	89.2%	
Borderline/Unknown	1972 (4.7%)	91.7%	
Subtype*			<0.001
Her2+/HR+	5017 (12.0%)	91.3%	
Her2+/HR-	2360 (5.7%)	84.7%	
Her2-/HR+	26661 (63.8%)	92.0%	
Triple Negative	5697 (13.6%)	74.3%	
Unknown	2022 (4.8%)	91.5%	

*Clinicopathological characteristics only for patients diagnosed after 2010 (N= 41757); **ER: estrogen receptor; PR: progesterone receptor; HER2: human epidermal growth factor receptor 2.

**Table 2:** Multivariate Cox regression analyses of patients

Variables	Multivariate analysis 1* (N= 142808)			Multivariate analysis 2** (N= 41757)		
	HR	95%CI	P value	HR	95%CI	P value
**Age**						
≤ 50	Reference			Reference		
51 - 60	1.026	0.990-1.064	0.157	0.972	0.883-1.070	.567
61 -70	1.195	1.150-1.242	<0.001	1.060	0.960-1.170	.253
> 70	1.847	1.781-1.915	<0.001	1.922	1.754-2.107	<0.001
**Race**						
Other	Reference			Reference		
White	1.086	1.052-1.121	<0.001	1.024	0.948-1.107	0.545
**Grade**						
Grade I	Reference			Reference		
Grade II	1.780	1.671-1.897	<0.001	1.857	1.530-2.254	<0.001
Grade III-IV	2.658	2.496-2.831	<0.001	3.469	2.865-4.202	<0.001
**Stage**						
I	Reference			Reference		
II	2.758	2.631-2.892	<0.001	3.791	3.179-4.521	<0.001
III	8.094	7.725-8.482	<0.001	12.831	10.800-15.243	<0.001
**ER Status**						
Positive	Reference			Reference		
Negative	1.297	1.245-1.351	<0.001	1.511	1.372-1.663	<0.001
Borderline/Unknown	1.062	0.953-1.183	0.277	1.369	0.794-2.359	0.259
**PR Status**						
Positive	Reference			Reference		
Negative	1.406	1.352-1.463	<0.001	1.966	1.785-2.166	<0.001
Borderline/Unknown	1.370	1.237-1.517	<0.001	1.457	0.900-2.361	0.126
**HER2 Status**						
Positive				Reference		
Negative				1.765	1.609-1.937	<0.001
Borderline/Unknown				1.417	1.138-1.764	0.002

*Multivariate analysis based on the patients of total cohort (N=142808); **Multivariate analysis based on the patients diagnosed after 2010; ***ER: estrogen receptor; PR: progesterone receptor; HER2: human epidermal growth factor receptor 2.

## Abbreviations

AJCC: American Joint Committee on Cancer
CDS: Conditional disease-specific survival
CS: Conditional survival
DSS: Disease-specific survival
ER: Estrogen receptor
Her2: human epidermal growth factor receptor 2
PR: Progesterone receptor
SEER: Surveillance, Epidemiology, and End Result
SD: Standard deviation
